# Association of genetic variants in ATR-CHEK1 and ATM-CHEK2 pathway genes with risk of colorectal cancer in a Chinese population

**DOI:** 10.18632/oncotarget.24299

**Published:** 2018-01-23

**Authors:** Shijia Wang, Yue Zhang, Min Chen, Yong Wang, Yifei Feng, Ziwei Xu, Dongsheng Zhang, Yueming Sun, Zan Fu

**Affiliations:** ^1^ The First School of Clinical Medicine, Nanjing Medical University, Nanjing, Jiangsu, China; ^2^ Department of General Surgery, The First Affiliated Hospital of Nanjing Medical University, Nanjing, Jiangsu, China

**Keywords:** colorectal cancer, genetic variants, *ATM*, susceptibility

## Abstract

**Objective:**

The *ATR-CHEK1* and *ATM-CHEK2* pathway have been confirmed to be related with the DNA damage response (DDR). Many studies have reported that genetic variants in ATR/CHEK1 and ATM/CHEK2 are associated with cancer risk. However, the association between genetic variants in ATR-CHEK1, ATM-CHEK2 pathway genes and colorectal cancer susceptibility is still unknown. In this study, we aim to explore whether these variants are correlated with the risk of colorectal cancer in a Chinese population.

**Methods:**

A hospital-based case-control study, including 1,121 cases and 1,056 controls was conducted to evaluate the association between eight selected single nucleotide polymorphisms (SNPs) (rs35514263 in *ATR*; rs492510, rs558351 in *CHKE1*; rs189037 in *ATM*; rs2236141, rs5762748, rs2236142 and rs9620817 in *CHEK2*) in ATR-CHEK1 and ATM-CHEK2 pathways and the risk of colorectal cancer in a Chinese population by using TaqMan method.

**Results:**

Individuals with rs189037 A allele were found to have a significantly increased risk of colorectal cancer, compared to those carrying G allele [odds ratio(OR) = 1.23, 95% confidence interval (CI) = 1.02–1.47 in dominant model and OR= 1.14, 95%CI= 1.01–1.29 in additive model]. And this risk is more pronounced in elder people (>69), rectum, early stage and poorly grade. In addition, bioinformatic analysis showed that rs189037 may change the secondary structure.

**Conclusions:**

Our results provide the evidence that rs189037 in *ATM* may increase the susceptibility of colorectal cancer in a Chinese population.

## INTRODUCTION

Colorectal cancer (CRC) is the third most common cancer and the fourth most common cause of death from cancer worldwide [1]. CRC has becoming a major health problem according to the evidence that more than a million of new cases of CRC are diagnosed per year worldwide and more than one-third of them result in death of cancer patients [2]. In the US, 142,820 estimated new cases and 50,830 cancer death occurred in 2013, which makes the colorectal cancer become the third leading cancer type [2]. And in China, new cases diagnosed with colorectal cancer in 2011 were 310,244, and the number of cancer deaths was 149,722 [3]. The development of CRC has been demonstrated as a complex process which was caused by many factors. Both environmental factors and genetic mutation play a vital role in the development of CRC [4]. Many studies reported that the inherited factors can influence the DNA repair capacity which may lead to the cancer development [5]. Therefore, individuals with inherited impairment in DNA repair capacity are often associated with increased risk of cancers [6].

Single nucleotide polymorphisms (SNPs) are the most familiar form of DNA variation which can influence gene expression and cellular functions [7, 8]. SNPs in different position have different functions. SNP in intronic region can regulate the transription of the gene and contribute to genetic susceptibility of the cancer [9]. SNP in the 5’UTR can impact promoter activity and transcription factor binding ability [10].

Genome-wide association studies (GWASs) have identified numerous genetic variants associated with the risk of the colorectal cancer [11–14]. Over 10 new colorectal cancer susceptibility loci were identified in East Asians [12, 15–17]. These studies provide additional insights into the genetic and biological basis of colorectal cancer.

The ataxia telangiectasia mutated (*ATM*) gene and ataxia telangiectasia and Rad3 related (*ATR*) gene which both belong to the *PI3Ks* family, are key to maintain chromosome integrity and genome stability [18–20]. ATR recognizes DNA single-strand breaks, which is damaged by UV radiation, and phosphorylates *CHEK1* (Ser345) to initiate cell cycle arrest and DNA replication inhibition [21–24]. The *ATM* gene, with the role of a damage recognition protein, is activated by DNA damage caused by ionizing radiation or reactive oxygen [25, 26]. After being phosphorylated by *ATM*, the *CHEK2* gene induces the transactivation of various proteins that function in cell-cycle arrest, apoptosis, DNA repair, and centrosome duplication [23]. Previous studies have shown that single-nucleotide polymorphisms (SNPs) of DNA repair genes affect not only individual risk for breast cancer [20, 27], but also lung cancer [28] and pancreatic cancer [29]. Zienolddiny et al found that the *ATR* T340C genotype was associated with a decreased risk of non–small cell lung cancer [30]. In contrast, the minor allele of rs6805118 was significantly associated with breast cancer risk for protective effect [20]. The SNPs rs521102 and rs2155388 in *CHEK1* were observed to be related with the increase incidence of pancreatic cancer and breast cancer, respectively [20, 29]. *ATM* polymorphisms rs664677 and rs609429, in the homozygote state were associated with increased breast cancer risk [27]. However, to our knowledge, no data has explored the association between *ATR-CHEK1* and *ATM-CHEK2* pathway genetic variants and CRC susceptibility in a Chinese population. We hypothesized that genetic variations in these pathways genes is related to the susceptibility of CRC. In this study, we conducted a case-control study to genotype the candidate SNPs in *ATR-CHEK1* and *ATM-CHEK2* pathway genes (rs35514263 in *ATR*; rs492510, rs558351 in *CHKE1*; rs189037 in *ATM*; rs2236141, rs5762748, rs2236142 and rs9620817 in *CHEK2*) and investigate the association with the risk of CRC in a Chinese population.

## RESULTS

### Characteristics of the study population

A total of 1,121 colorectal cancer patients and 1,056 controls were recruited in this study and the distributions of selected characteristics of the cases and controls are summarized in Table 1. No statistical differences were found between cases and controls for age, sex and smoking status (*P* = 0.200, 0.140, and 0.200, respectively). Among the CRC cases, 49.6% of patients suffered from colon cancer, while 50.4% from rectum cancer. In terms of histologic differentiation, 5.44%, 68.15%, and 26.41% of CRCs were grouped as low grade, intermediate grade, and high grade, respectively. The Dukes A, B, C, and D stages were 9.46%, 39.96%, 34.79%, and 15.79%, respectively.

**Table 1:** Distribution of selected variables between colorectal cancer cases and controls

		Cases (n=1121)		Controls (n=1056)		P^a^
		N	%	N	%	
Age (years) mean ± SD		60.2±12.4		59.4±17.7		0.20
Sex	Male	631	56.29%	561	53.12%	0.14
	Female	490	43.71%	495	46.88%	
Smoking status	No	794	70.83%	775	73.39%	0.20
	Yes	327	29.17%	281	26.61%	
Tumor site	Rectum	565	50.40%			
	Colon	556	49.60%			
Dukes stage	A	106	9.46%			
	B	448	39.96%			
	C	390	34.79%			
	D	177	15.79%			
Tumor grade	Low	61	5.44%			
	Intermediate	764	68.15%			
	High	296	26.41%			

^a^ Two-sided Student's *t*-test or x^2^ test.

### Associations of selected SNPs and CRC risk

The frequency distributions of all SNPs and the risk of CRC for cases and controls are listed in Table 2. The genotype distributions of eight SNPs in the control group were in accordance with the Hardy-Weinberg equilibrium (HWE) which meant there was no selection bias (*P* = 0.860 for rs35514263, *P* = 0.856 for rs492510, *P* = 0.400 for rs558351, *P* = 0.280 for rs189037, *P* = 0.275 for rs2236141, *P* = 0.406 for rs5762748, *P* = 0.941 for rs2236142 and *P* = 0.837 for rs9620817, respectively). We observed that only rs189037 in *ATM* were significantly associated with CRC susceptibility. The genotype frequencies of rs189037were 30.4% (GG), 49.1% (GA), and 20.5% (AA) in cases, which were statistically different from that in the control group (34.7% GG, 47.0% GA, and 18.3% AA) (*P*=0.0297 in additive model, *P*=0.026 in dominant model). After adjustment for age, sex and smoking statue, we found that variant genotypes of rs189037 were significantly associated with the increased risk of CRC (adjusted OR = 1.14, 95%CI = 1.01-1.29 in additive model; adjusted OR = 1.23, 95%CI =1.02-1.47 in dominant model, respectively). And no significant association was found between rs35514263, rs492510, rs558351, rs2236141, rs5762748, rs2236142, rs9620817 and CRC susceptibility.

**Table 2:** Association between the selected tagSNPs and CRC risk

SNPs	Allele^a^	Cases^b^ (n=1121)	Controls^b^ (n=1056)	MAF^c^ (case/control)	P_HWE_^d^	Adjusted OR(95%CI)^e^			P^f^
						Additive model	Dominant model	Recessive model	
rs35514263	C/T	617/208/22	677/200/14	0.148/0.128	0.860	1.19(0.98-1.44)	1.18(0.95-1.47)	1.66(0.85-3.27)	0.132
rs492510	A/G	329/529/255	269/521/258	0.467/0.495	0.856	0.90(0.80-1.02)	0.83(0.69-1.00)	0.92(0.75-1.12)	0.086
rs558351	C/T	326/397/133	350/437/121	0.387/0.374	0.400	1.06(0.92-1.21)	1.02(0.84-1.23)	1.20(0.92-1.57)	0.861
**rs189037**	G/A	336/543/227	362/491/191	0.451/0.418	0.280	**1.14(1.01-1.29)**	**1.23(1.02-1.47)**	1.15(0.93-1.43)	**0.028**
rs2236141	C/T	600/227/22	630/243/30	0.159/0.168	0.275	0.94(0.79-1.13)	0.96(0.78-1.18)	0.77(0.44-1.35)	0.673
rs5762748	G/A	632/167/14	687/174/8	0.120/0.109	0.406	1.11(0.90-1.38)	1.09(0.86-1.37)	1.82(0.76-4.37)	0.489
rs2236142	G/C	308/423/115	317/431/148	0.386/0.406	0.941	0.92(0.80-1.06)	0.96(0.79-1.16)	0.80(0.61-1.04)	0.658
rs9620817	A/T	710/141/5	752/149/8	0.088/0.091	0.837	0.97(0.77-1.22)	0.99(0.77-1.26)	0.66(0.22-2.03)	0.913

Abbrevations: OR, odds ratio; CI, confidence interval.

^a^Major/minor.

^b^Numbers of major homozygote/heterozygote/minor homozygote.

^c^Minor allele frequency in cases/controls.

^d^HWE, Hardy-Weinberg equilibrium in control subjects.

^e^Adjusted for age, sex, smoking and drinking status in logistic regression model.

^f^P for dominant model.

### Stratification analysis of associations with CRC

We further conducted the stratification analyses by age, sex and smoking statue to detect weather these confounders played roles in the CRC risk. The results are shown in Table 3. The increased risk associated with rs189037 was significant in elder group (age>69) (adjusted OR = 1.43, 95%CI = 1.01-2.02, *P* = 0.045). And sex and smoking statue were not the potential confounders (*P* = 0.256 in males, *P* = 0.060 in females, *P* = 0.198 in smokers, *P* = 0.070 in non-smokers, respectively).

**Table 3:** Stratification analyses between rs189037 genotypes and CRC risk

Variables	Case/control N	Genotype (cases/controls)				Adjusted OR (95%CI)^a^	P^b^
		GG		GA/AA			
		N	%	N	%		
Age (years)							
≤69	839/728	251/235	29.9/32.3	588/493	70.1/67.7	1.13(0.91-1.40)	0.283
>69	267/316	85/127	31.8/40.2	182/189	68.2/59.8	**1.43(1.01-2.02)**	**0.045**
Sex							
Male	622/554	195/189	31.4/34.7	427/365	68.6/65.9	1.15(0.90-1.48)	0.256
Female	484/490	141/173	29.1/35.3	343/317	70.9/64.7	1.30(0.99-1.70)	0.060
Smoking status							
Yes	320/277	107/106	33.4/38.3	213/171	66.6/61.7	1.25(0.89-1.76)	0.198
No	786/767	229/256	29.1/33.4	557/511	70.9/66.6	1.22(0.98-1.51)	0.070

^a^OR (odds ratio), CI (confidence interval).

^b^P values were calculated in dominant model with adjustment for age, sex and smoking status.

### Associations between rs189037 and clinicopathologic parameters of CRC

The subgroup analysis was further performed to evaluate the association between rs189037 polymorphism and clinicopathological characteristics of CRC (Table 4). We observed that rs189037 GA/AA genotypes were associated with an increased colorectal cancer risk in individuals with rectal cancer (OR = 1.30, 95% CI = 1.05-1.64), poor-differentiated CRC (OR = 1.32, 95% CI = 1.08-1.61), and early stage cancer (Dukes A and B) (OR = 1.27, 95% CI = 1.01-1.59). No significant difference was observed in other subgroup.

**Table 4:** Associations between the rs189037 polymorphism and clinicopathologic parameters of CRC

Variables	GG		GA/AA		GA/AA vs GG	P^b^
	N	%	N	%	Adjusted OR (95%CI)^a^	
Cases (n=1106)	336	30.4	770	69.6		
Controls (n=1044)	362	34.7	682	65.3		
Dukes stage						
A+B	163	29.7	385	70.3	**1.27(1.01-1.59)**	**0.037**
C+D	173	31.0	385	69.0	1.19(0.95-1.48)	1.127
Tumor site						
Colon	175	31.7	377	68.3	1.15(0.92-1.43)	0.215
Rectum	161	29.1	393	70.9	**1.31(1.05-1.64)**	**0.019**
Tumor grade						
Poor--differentiated	235	28.9	578	71.1	**1.32(1.08-1.61)**	**0.007**
Well-differentiated	101	34.5	192	65.5	1.00(0.76-1.31)	0.990

^a^OR (odds ratio), CI (confidence interval).

^b^P values were calculated in dominant model with adjustment for age, sex and smoking status.

### Prediction of rs189037 on *ATM* folding structures

**Figure 1:**
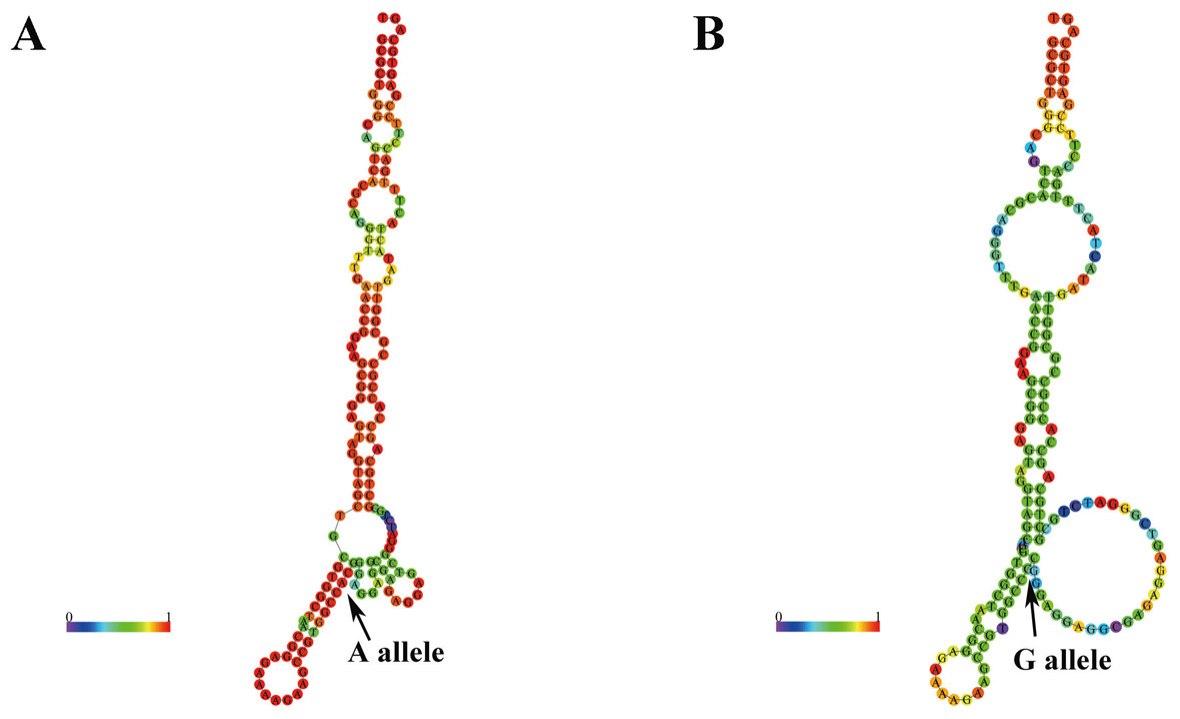
Prediction of rs189037 on *ATM* folding structure Arrow, which indicated the position of rs189037, showed the secondary structure change caused by rs189037. Arrow A indicates the sequences of A allele, whereas arrow G indicates the G allele. These structures were predicted by inputting two 80-nt long *ATM* DNA sequences centering the rs189037 locus into RNAfold (http://rna.tbi.univie.ac.at), with **(A)** the rs189037-A or **(B)** rs189037-G.

We used RNAfold to predict the *ATM* secondary structure of selected SNPs. We found the secondary structure of rs189037 G/A alleles (Figure 1), rs558351 C/T alleles, and rs9620817 A/T alleles were significant changed (Supplementary Figures 2 and 6). However, there was a little change observed in other SNPs except rs492510 A/G alleles (Supplementary Figures 1, 3, 4, 5 and 7).

## DISCUSSION

In this case-control study, we evaluated the association of genetic variants in *ATR-CHEK1* and *ATM-CHEK2* genes and susceptibility of CRC in a Chinese population. Eight SNPs were selected and rs189037 in *ATM* was found to be associated with CRC risk. It was obvious that rs189037 allele A increased the CRC risk.

Accumulated evidence demonstrated that *ATM* protein sensed DNA double strand breaks (DSB) caused by either genetically programmed or the appearance of selected exogenous factor, and then activates *CHEK2* to initiate cell cycle arrest and DNA replication inhibition [19, 31–34]. The SNP rs189037 was a common polymorphism in the promoter region of *ATM* gene which was able to affect expression of *ATM* mRNA by differentially binding to AP-2α and further influence *ATM* protein activity [35, 36]. Considering that rs189037 may regulate the *ATM* expression, it is meaningful for us to evaluate its association with cancer risk [35]. Several studies have examined the association of rs189037 with kinds of cancer susceptibility. Liu et al confirmed that individuals carrying variant AA genotype of rs189037 had higher lung cancer risk than those carrying GG genotype [37]. In addition, the G allele of ATM rs189037 exhibited a protective effect against thyroid carcinoma [38]. While rs189037 was not found to be associated with breast cancer, glioma or leukemia [39–42]. To the best of our knowledge, it was the first time that we found that rs189037 was associated with susceptibility of CRC in a Chinese population.

Some environmental factors, such as alcohol intake and tobacco smoking, were related with tumorigenesis [43–45]. Our stratification analyses demonstrated that individuals carrying GA/AA genotype whether smoking or not had not significantly increased the risk of colorectal cancer which not consist with published studies [45]. It is important to note that the limited sample size in smokers subgroups didn't have sufficient statistical power to confirm our conclusion. More studies may be conducted to confirm if GA/AA genotype of rs189037 increasing CRC risk could partly attribute to the accumulated exposure/exposure history to tobacco carcinogens. Long-term alcohol intake was associated with increased CRC risk [43, 44]. However, due to the serious lacking the data of alcohol intake, we did not do this stratification analyses which we would complete in the future. Moreover, we found that increased colorectal cancer risk correlated with rs189037 was more significant in subgroup of elder individuals which suggested that promoting effects of *ATM* variants on colorectal cancer may be modulated by specific epidemiological features. However, we found there was no association between colorectal cancer and sex. Similarly, because of the lacking of data of cancer family history, we did not analyze the relationship between family history and colorectal cancer tumorigenesis. The results above strengthened the conclusion that the development of CRC was a complex process caused by environmental factors and genetic mutation. However, further studies are needed to confirm these results.

Furthermore, we found that rs189037 GA/AA genotype increased the risk of rectum cancers and poor-differentiated CRC. In addition, the individuals with GA/AA genotype associated with colorectal cancer among patients with Duke's stage of A or B which demonstrated rs189037 genetic variant played a role in the early stage of cancer. And this result consisted with published studies that *ATM* had a role in the early stage of colorectal cancer development [46]. We could draw the conclusion that carcinogenesis of colorectal cancer of different site and grade regulated by different molecular biological mechanisms.

And next, we used RNAfold to predict the secondary structure changes caused by SNPs. We found the genetic variant of rs189037, rs558351 and rs9620817 may markedly change the folding architectures. By using HaploReg v4.1 [47], we found rs189037 could alter 10 motifs, including BCL_disc9, CHD2_disc3, E2F_disc3, ELF1_disc3, Ets_disc9, Myc_disc10, NRSF_disc9, Rad21_disc8, SZF1-1 and Znf143_disc4. Some of them have been confirmed to be associated with carcinogenesis [48, 49]. In addition, rs189037 belongs to the eQTL of *ACAT1* and *ATM*, which have been reported to be associated with carcinogenesis of colorectal cancer [50], so we speculate this genetic variant may increase the colorectal cancer susceptibility by influencing their expression. And considering the fact that genetic variation rs189037 is located in 5’UTR of *ATM* gene and several studies have confirmed that the genetic variant can impact promoter activity and transcription factor binding ability [10], we speculated that the genetic variation may lead to an alteration of *ATM* expression and affect the mRNA binding process and thus are associated with colorectal cancer susceptibility. However, results above were just our inferences, it is necessary to test their authenticity in future studies.

In conclusion, we demonstrated that rs189037 in *ATM* was associated with increased CRC risk in a Chinese population. In addition, in the stratified analyses, increased risk was found to be more pronounced in older people, people diagnosed with rectal cancer, and patients with Duke's A/B stage or poor-differentiated tumor grade. Further validation of large population-based studies in different ethnicities is still needed.

## MATERIALS AND METHODS

### Ethics statement

The study was approved by the institutional review board of Nanjing Medical University. Informed written consent was obtained from all subjects. In addition, the experimental methods were carried out in accordance with the approved guidelines.

### Study participants

The characteristics of CRC patients and controls in this study are described in Table 1. We recruited 1,121 patients with CRC and 1,056 cancer-free controls without age or sex restrictions at the First Affiliated Hospital of Nanjing Medical University, Nanjing, China. All the patients were histologically confirmed. The cancer-free control patients were randomly selected from emergency of the same hospital during the same period, none of them had self-reported history of cancers or colorectal polyps. The controls were matched by age and sex to the CRC patients. About 5 mL of venous blood sample was collected from each subject after obtaining a written informed consent.

### SNP selection

**Figure 2:**
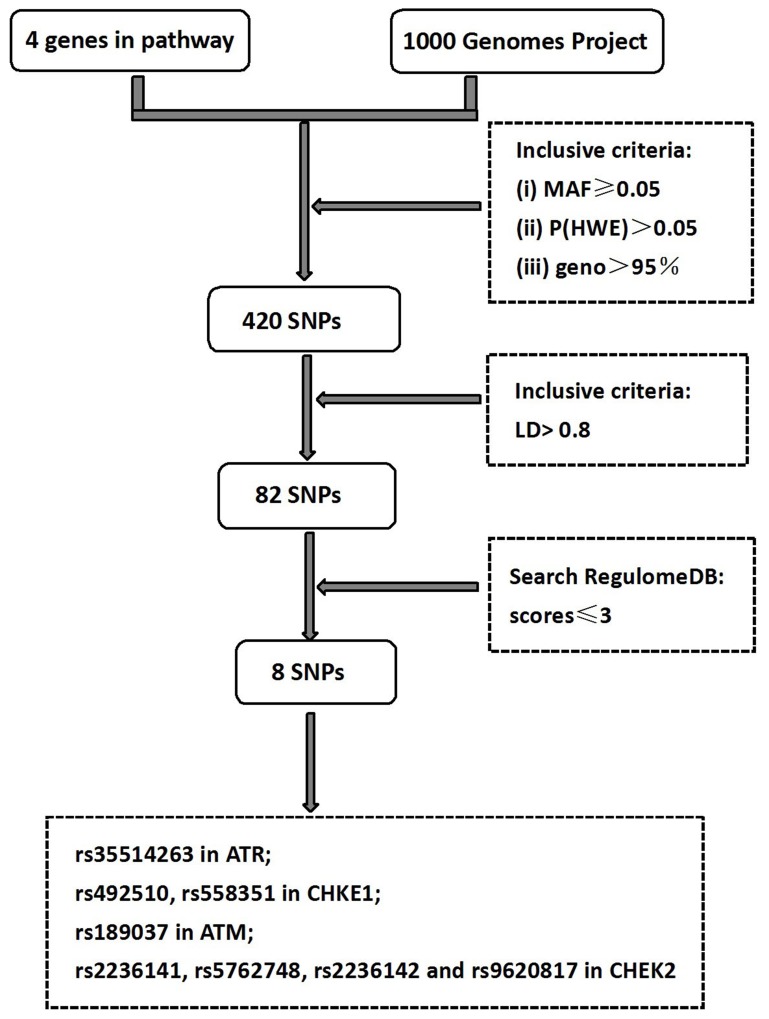
Schematic flow for searching the tagSNPs in *ATR/CHEK1, ATM/CHEK2*

The selection process of the genes and SNPs is shown in Figure 2. Firstly, the 1000 Genomes Projects was used to search all SNPs (Chinese Han population) in respective gene region (including 2 kb up-stream region of each gene). A total of 420 SNPs in ATR-CHEK1 and ATM-CHEK2 pathways genes were selected which all fit the following there criteria: (i) minor allelic frequency (MAF) ≥0.05 ; (ii) *P* (HWE)>0.05; (iii) geno call rate >95%. Secondly, 338 SNPs were excluded because it does not match the criteria of linkage disequilibrium (LD) >0.8 by using the HaploView 4.2 software. The potentially functional SNPs with scores ≤3 were marked by Search RegulomeDB (http://regulome.stanford.edu/index), which has potential functions of protein structure, gene regulation, splicing and microRNA (miRNA) binding, with consideration of whether the alternative alleles of a SNP were likely to have differential effects on gene function. As a result, a total of 8 SNPs (rs35514263 in *ATR*; rs492510, rs558351 in *CHKE1*; rs189037 in *ATM*; rs2236141, rs5762748, rs2236142 and rs9620817 in *CHEK2*) were finally chosen for genotyping.

### Genotyping

The 384-well ABI 7900HT Real-Time PCR System (Applied Biosystems, Foster City, CA, USA) was used as the TaqMan SNP Genotyping assay. The reaction conditions were set as follows: 95 °C for 10 min followed by 40 cycles of 95 °C for 15 sec, and60 °C for 1 min. And SDS2.4 software (Applied Biosystems) was used to read and analyze allelic discrimination. The average call rates for eight SNPs were more than 95%. We also randomly selected over 10% of the samples for repeated assays to assess the reproducibility and the concordance rate was 100%.

### Statistical analysis

The Hardy-Weinberg equilibrium (HWE) of the controls’ genotype frequencies was evaluated by a goodness-of-fit chi-square test (χ^2^ test). Differences in the distribution of epidemiological variables between cases and controls were evaluated by the χ^2^ test for categorical variables and student's *t*-test for continuous variables. Odds ratios (ORs) and their 95% confidence intervals (CIs) were using to examine the correlation between different genotypes and colorectal cancer risk by univariate and multivariate logistic regression models. Age, sex and smoking status were the possible confounders. All *P*-values presented were two-sided and were considered statistically significant at *P* <0.05. Statistical analyses were conducted using Statistical Analysis System (SAS) software (version 9.4; SAS Institute Inc, Cary, NC, USA).

### Prediction of secondary structures

We used RNAfold (http://rna.tbi.univie.ac.at/) to predict the folding structure variants of *ATM* on account of tagSNPs genotypes [51].

## SUPPLEMENTARY MATERIALS FIGURES


