# Supplementary material for: Association of genetic variants in ATR-CHEK1 and ATM-CHEK2 pathway genes with risk of colorectal cancer in a Chinese population
Source: Oncotarget. 2018 Jan 23;9(42):26616–24. doi: 10.18632/oncotarget.24299 (PMC6003554; doi:10.18632/oncotarget.24299)
Supplement: Supplementary file 1 [file oncotarget-09-26616-s001.pdf]

Association of genetic variants in ATR-CHEK1 and ATM-CHEK2 pathway genes with risk of colorectal cancer in a Chinese population

SUPPLEMENTARY MATERIALS

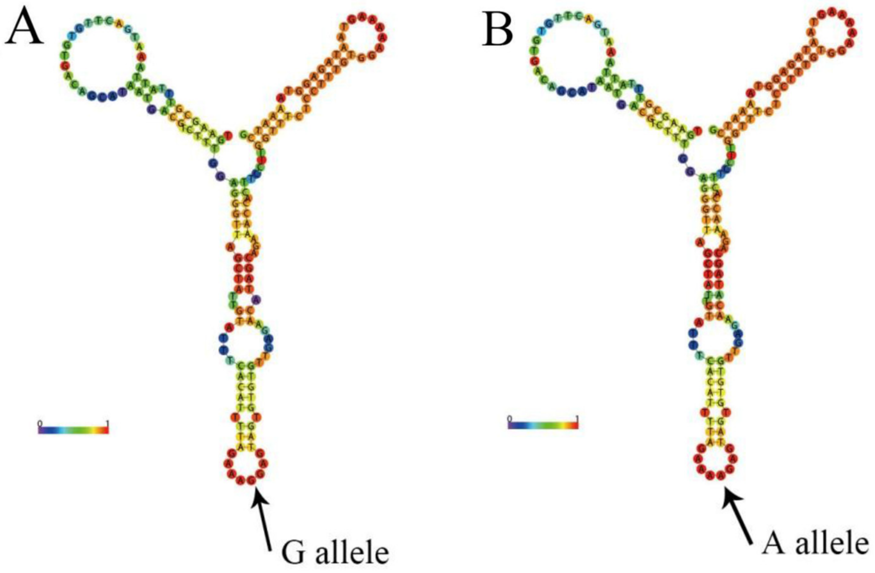

Supplementary Figure 1: Prediction of rs492510 on *CHEK1* folding structure. Arrow G indicates the sequences of G allele, whereas arrow A indicates the A allele. These structures were predicted with **(A)** the rs492510-G or **(B)** rs492510-A.

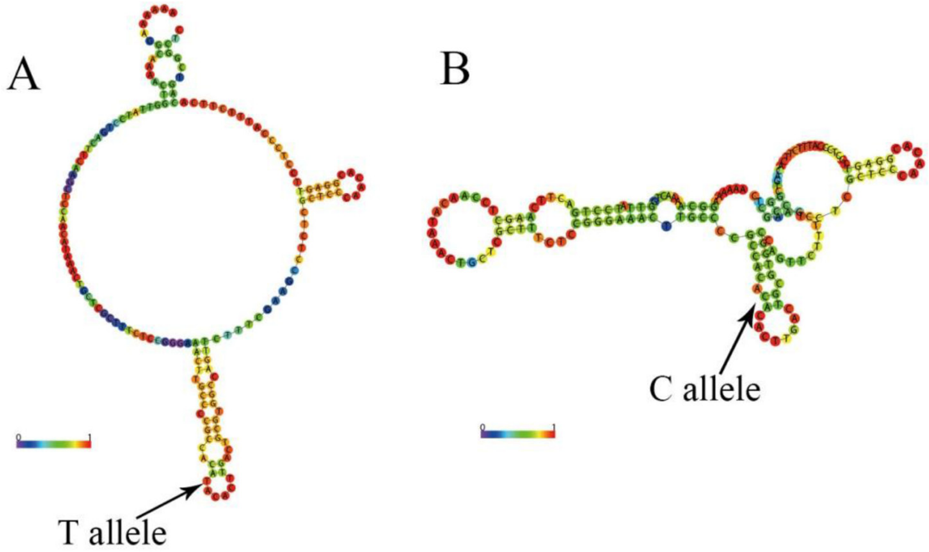

Supplementary Figure 2: Prediction of rs558351 on *CHEK1* folding structure. Arrow T indicates the sequences of T allele, whereas arrow C indicates the C allele. These structures were predicted with **(A)** the rs558351-T or **(B)** rs558351-C.

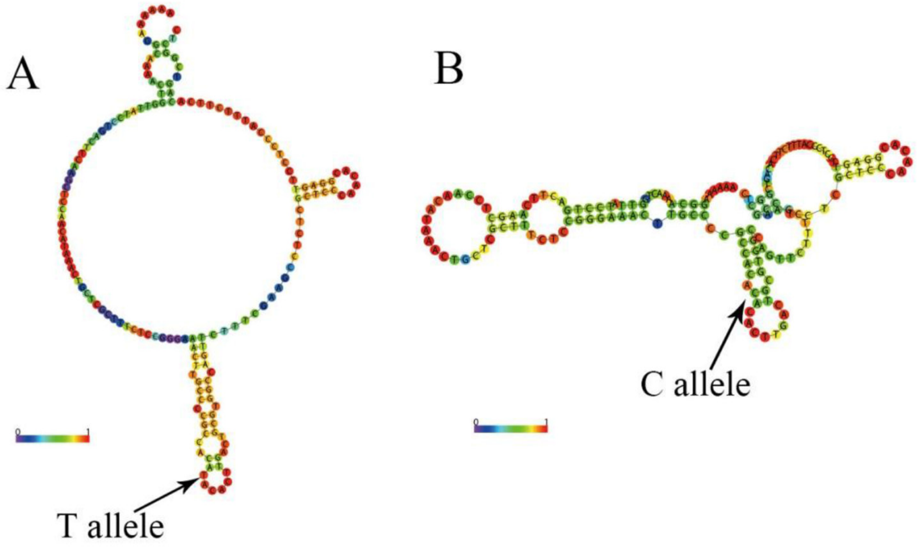

Supplementary Figure 3: Prediction of rs2236141 on *CHEK2* folding structure. Arrow T indicates the sequences of T allele, whereas arrow C indicates the C allele. These structures were predicted with **(A)** the rs2236141-T or **(B)** rs2236141-C.

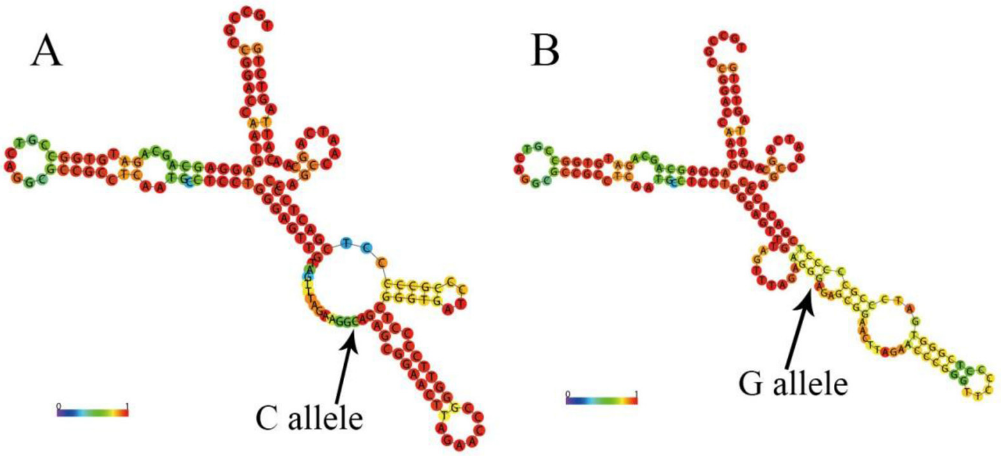

Supplementary Figure 4: Prediction of rs2236142 on *CHEK2* folding structure. Arrow C indicates the sequences of C allele, whereas arrow G indicates the G allele. These structures were predicted with **(A)** the rs2236142-C or **(B)** rs2236142-G.

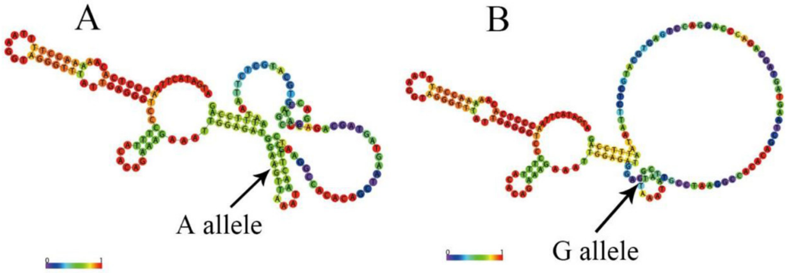

Supplementary Figure 5: Prediction of rs5762748 on *CHEK2* folding structure. Arrow G indicates the sequences of G allele, whereas arrow A indicates the A allele. These structures were predicted with **(A)** the rs5762748-A or **(B)** rs5762748-G.

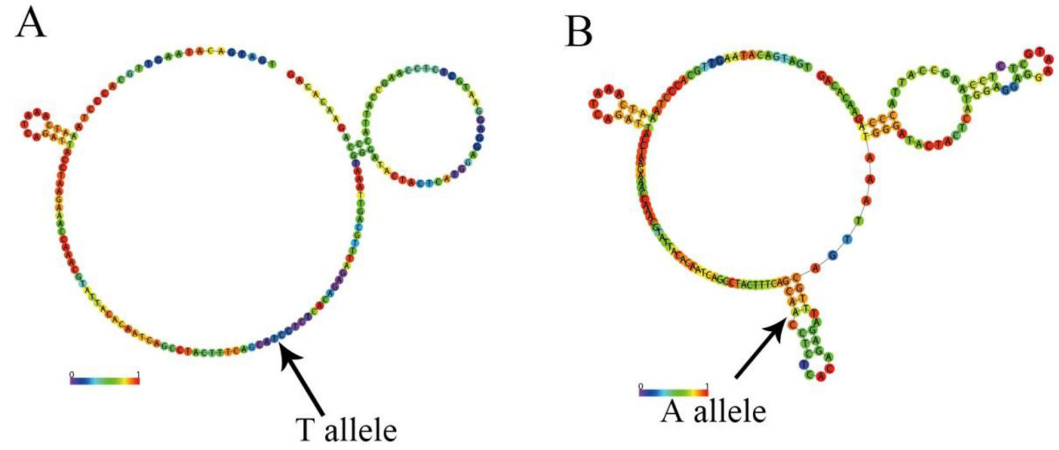

Supplementary Figure 6: Prediction of rs9620817 on *CHEK2* folding structure. Arrow T indicates the sequences of T allele, whereas arrow A indicates the A allele. These structures were predicted with **(A)** the rs9620817-T or **(B)** rs9620817-A.

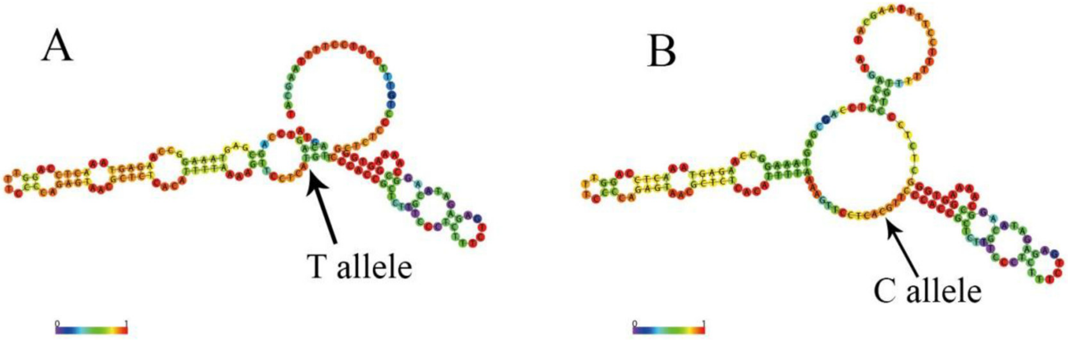

Supplementary Figure 7: Prediction of rs35514263 on *ATR* folding structure. Arrow T indicates the sequences of T allele, whereas arrow C indicates the C allele. These structures were predicted with **(A)** the rs35514263-T or **(B)** rs35514263-C.
